# Potential clinical implications due to essential differences in antimicrobial susceptibility profiles of actinomycetoma causative agents of the *Actinomadura*, *Nocardia* and *Streptomyces* genera towards commonly used anti-actinomycetoma drugs

**DOI:** 10.1093/jac/dkag325

**Published:** 2026-09-25

**Authors:** Rihab A H Mohammed, Andrew K Watson, Mickey Konings, Katarzyna Mickiewicz, Ahmed H Fahal, Alexandro Bonifaz, Doudou Sow, Jeff Errington, Michiel L Bexkens, Wendy W J van de Sande

**Affiliations:** Erasmus MC, Department of Medical Microbiology and Infectious Diseases, University Medical Center Rotterdam, Rotterdam, The Netherlands; Department of Medical Microbiology and Parasitology, Faculty of Medical Laboratory Sciences, University of Dongola, Dongola, Sudan; Mycetoma Research Centre, University of Khartoum, Khartoum, Sudan; Centre for Bacterial Cell Biology, Bioscience Institute, Newcastle University, Newcastle upon Tyne, UK; Erasmus MC, Department of Medical Microbiology and Infectious Diseases, University Medical Center Rotterdam, Rotterdam, The Netherlands; Centre for Bacterial Cell Biology, Bioscience Institute, Newcastle University, Newcastle upon Tyne, UK; Mycetoma Research Centre, University of Khartoum, Khartoum, Sudan; Hospital General de México Dr Eduardo Liceaga: Ciudad de Mexico, Mexico City, Mexico; Department of Parasitology-Mycology, UFR Sciences de La Santé, Université Gaston Berger, Saint Louis, Senegal; Centre for Bacterial Cell Biology, Bioscience Institute, Newcastle University, Newcastle upon Tyne, UK; Sydney Infectious Diseases Institute, Faculty of Medicine and Health, University of Sydney, Sydney, Australia; Erasmus MC, Department of Medical Microbiology and Infectious Diseases, University Medical Center Rotterdam, Rotterdam, The Netherlands; Erasmus MC, Department of Medical Microbiology and Infectious Diseases, University Medical Center Rotterdam, Rotterdam, The Netherlands

## Abstract

**Background:**

Actinomycetoma is a neglected tropical disease characterized by large tumorous lesions. It is caused by actinomycetes belonging to the genera *Actinomadura*, *Nocardia* and *Streptomyces.* Despite the differences between these genera and species, all actinomycetoma cases are treated with the same antibiotics. It is currently not known if the actinomycetoma causative agents have similar susceptibility patterns against these antibiotics. We therefore set out to determine the MICs for the most common causative agents against commonly used antibiotics.

**Materials and methods:**

We used the standard *in vitro* susceptibility method from the CLSI to assess the antimicrobial susceptibility of 94 actinomycete isolates belonging to the *Actinomadura*, *Nocardia* and *Streptomyces* genera and associated with actinomycetoma against 12 antibiotics. The molecular basis behind the reduced susceptibility was further explored using the genomes of the isolates.

**Results:**

All isolates were highly susceptible to linezolid (100%), amikacin (98.9%) and trimethoprim/sulfamethoxazole (87.2%). For amoxicillin/clavulanate and rifampicin, species-dependent susceptibility was noted. We found that 55% of *Actinomadura madurae* isolates, 61.5% of *Nocardia brasiliensis* isolates and 100% of *Streptomyces somaliensis* and *Streptomyces sudanensis* were susceptible to amoxicillin/clavulanate. All *A. madurae*, *Actinomadura pelletieri* and *N. brasiliensis* isolates were resistant to rifampicin, whereas all *S. somaliensis* and *S. sudanensis* isolates were susceptible. This was most likely due to amino acid differences in the RpoB gene.

**Conclusions:**

Clear differences in antimicrobial susceptibility patterns were noted for the different actinomycetoma causative agents, indicating that actinomycetoma treatment should be tailored to the causative agent.

## Introduction

Actinomycetoma is a neglected tropical disease that primarily affects individuals in tropical and subtropical regions.^1^ It is characterized by large subcutaneous lesions, most often on the feet.^2^ The disease starts with the traumatic inoculation of the causative agent, typically through punctures from thorns or other mechanical means^1^

Over the past few decades, various actinomycetoma causative agents have been identified, but the most common are *Nocardia brasiliensis*, *Streptomyces somaliensis*, *Streptomyces sudanensis*, *Actinomadura madurae* and *Actinomadura pelletieri.*^1^ Of these, *N. brasiliensis* is mainly confined to Latin America, while *A. madurae*, *A. pelletieri*, *S. somaliensis* and *S. sudanensis* are mainly found in Africa.^1^

Despite the diversity of actinomycetoma agents, the currently used standard treatment is dependent on the experience of the treating physician and not on the causative agent. Since the early 1960s, actinomycetoma has been treated with trimethoprim/sulfamethoxazole. Trimethoprim/sulfamethoxazole alone will result in a cure rate of approximately 60% following prolonged treatment.^3,4^ Therefore, other antibiotics are often used in combination with trimethoprim/sulfamethoxazole. Since 1982 amikacin has been used in combination treatments with trimethoprim/sulfamethoxazole in Latin America.^5^ This treatment is referred to as the ‘Welsh regimen’ and consists of five weekly cycles of 8/40 mg/kg/day trimethoprim/sulfamethoxazole for 5 weeks and 15 mg/kg IV amikacin during the first 3 weeks of this cycle.^6^ In other regions, other antibiotics, such as amoxicillin/clavulanate, tetracycline, penicillin and, more recently, linezolid, have also been employed in combination with trimethoprim/sulfamethoxazole.^7–10^ Most of these treatment regimens were originally developed in Mexico, where *N. brasiliensis* is the main causative agent, and were subsequently implemented for the treatment of actinomycetoma caused by other causative agents without *in vitro*, *in vivo* or clinical data to back this up. However, actinomycetoma lesions caused by *Actinomadura* species and *S. somaliensis* differ from those caused by *N. brasiliensis*. The grains—the biofilm structures in which the causative agents reside in the human tissue—in the *Streptomyces* and *Actinomadura* lesions are often bigger compared to *Nocardia*.^11^ Furthermore, clinical observations indicate that treatment failures in actinomycetoma cases caused by *A. madurae* with the Welsh regimen are more common than in actinomycetoma cases caused by *N. brasiliensis.*^11^ In Sudan, therefore, the most common treatment for these three species is trimethoprim/sulfamethoxazole combined with amoxicillin/clavulanate.^12^

Due to the lack of susceptibility data for the different actinomycetoma causative agents, the reasons behind these differences in treatment responses are currently not known. A direct comparison between the *in vitro* susceptibilities of the different actinomycetoma causative agents to the most commonly used antibiotics in actinomycetoma treatment has not been made. This lack of information presents challenges for clinicians aiming to optimize patient management. Therefore, in this study we adapted the CLSI M24 guidelines for antimicrobial susceptibility testing to determine the susceptibilities of *Actinomadura*, *Nocardia* and *Streptomyces* species implicated as actinomycetoma causative agents. We also used genomic comparisons to see if there were genetic mutations that could explain the resistance noted.

## Materials and methods

### Bacterial isolates

In this study we assessed the antimicrobial susceptibilities against 94 different *Actinomadura*, *Nocardia* and *Streptomyces* isolates. The reference isolates were obtained from the Deutsche Sammlung von Mikroorganismen und Zellkulturen, Braunschweig, Germany (DSM) collection (*n* = 34), from the Westerdijk Fungal biodiversity Institute, Utrecht, The Netherlands (CBS) collections (*n* = 6) and the ATCC, Manassas, VA, USA (*n* = 1). Clinical isolates were obtained from the Mycetoma Research Center (MRC), University of Khartoum, Sudan (*n* = 39); from Hospital General de México Dr Eduardo Liceaga, Mexico (*n* = 11); from Université Gaston-Berger (UGB), Senegal (*n* = 2); and from Erasmus Medical Center (EMC) (*n* = 1) (Table 1; and Table S1, available as Supplementary data at *JAC* Online). All species were previously molecularly identified by either sequencing the 16S rRNA or heat-shock protein 65 (HSP65) barcoding genes or Whole Genome Sequencing.^13^ All actinomycetes were cultured on trypticase soy agar (TSA) supplemented with 5% sheep blood (BD BBL^TM^ stackerplate; ref. 254087) for 7–14 days depending on the species, as indicated in Table 1.

**Table 1.** Isolates used

Organism	*n* isolates used	Common actinomycetoma causative agent^1^	Culture time TSA, days	Incubation time MIC, days
*Actinocorallia weidayensis*	1	No	7	7
*Actinomadura cremea*	1	No	7	7
*Actinomadura geliboluensis*	2	Rare	7	7
*Actinomadura hallensis*	1	No	7	7
*Actinomadura latina*	1	Rare	7	7
*Actinomadura madurae*	20	Common	7	7
*Actinomadura meyerae*	2	No	7	7
*Actinomadura pelletieri*	12	Common	14	7–10
*Actinomadura welshii*	2	Rare	7	7
*Nocardia asteroides*	1	Occasional	7	4
*Nocardia brasiliensis*	13	Common	7	4
*Nocardia farcinica*	1	Rare	7	4
*Nocardia mexicana*	1	Rare	7	4
*Nocardia vulneris*	2	Rare	7	4
*Streptomyces albidoflavus*	2	No	7	4
*Streptomyces albogriseolus*	1	No	7	4
*Streptomyces chrestomyceticus*	1	No	7	4
*Streptomyces griseus*	1	Rare	7	4
*Streptomyces kordofanensis*	1	Rare	7	4
*Streptomyces radiopugnans*	1	Rare	7	4
*Streptomyces somaliensis*	7	Common	7	4
*Streptomyces sudanensis*	19	Rare	7	4
*Streptomyces werraensis*	1	Rare	7	4

TSA, trypticase soy agar.

### Antibiotics

The antimicrobial susceptibilities were determined against the following 12 antimicrobial agents: amoxicillin/clavulanate, amikacin, dapsone, doxycycline, gentamicin sulphate, linezolid, meropenem, minocycline, moxifloxacin, netilmicin, rifampicin and trimethoprim/sulfamethoxazole. Respective solvents, brands and tested concentration ranges are stated in Table S2. Antibiotic stock solutions were diluted 1:200 in Mueller–Hinton (MH) broth, and a 2-fold dilution range was prepared in a 96-well plate.

### *In vitro* susceptibility testing

*In vitro* susceptibility testing was conducted according to M24 CLSI guidelines.^14^ For *A. madurae* and the majority of *Actinomadura* species, *Nocardia* species and *Streptomyces* species the MIC was read after a 72 h incubation at 37°C. For *A. pelletieri* the MIC was read after 120 h. *Nocardia asteroides* ATCC19247 and *Staphylococcus aureus* ATCC29213 were used as the quality control strains.

### Investigating the molecular basis of resistance

For all phylogenies, sequences were aligned using MAFFT with the –auto flag,^15^ alignments were trimmed using trimAl with the -automated1 flag,^16^ and all phylogenies were inferred using iqtree2 (version 2.3.6) with the settings –alrt 1000 -B 1000 -m TEST.^17,18^ 16S rRNA sequences were derived either from Sanger sequencing where available, or from identification of 16S rRNA sequences from whole genomes using CMsearch^19^ with the RFAM bacterial SSU rRNA family (RF00177) as a query.^20^ Protein sequences from all isolates were annotated using eggnog-mapper emapper (version 2.1.13), with eggnog family definitions used to identify RpoB, ROX, *folA*, *folC* and *folP* homologues.^21^ For ROX, *folA*, *folC* and *folP*, a phylogeny of all members of the broader eggnog family was generated, with only the clan of genes closely related to charactered reference sequences from *Nocardia* considered. In the case of ROX, BLAST search against the nr database was used to confirm the best hits for these selected sequences which were subsequently annotated as rifampin monooxygenase.^22^

Motif logos were generated using LogoMaker.^23^ The crystal structure of *E. coli* RpoB in complex with rifampicin was downloaded from the Protein Data Bank (PDB). Crystal structures of RpoB from actinomycetoma isolates were predicted using AlphaFold Server.^24^ Crystal structures were aligned and visualized using pymol (PyMOL Molecular Graphics System, Version 3.0; Schrödinger, LLC).

AMRFinderPlus (version 4.2.7) was run in combined mode, taking advantage of both genome sequences and prebuilt annotations, with the addition of the –plus flag,^25^ and the results were used to explore the distribution of known resistance genes (e.g. β-lactamases) across isolates. All data are also available at Figshare (doi:10.6084/m9.figshare.33406600).

## Results

**Figure 1.**
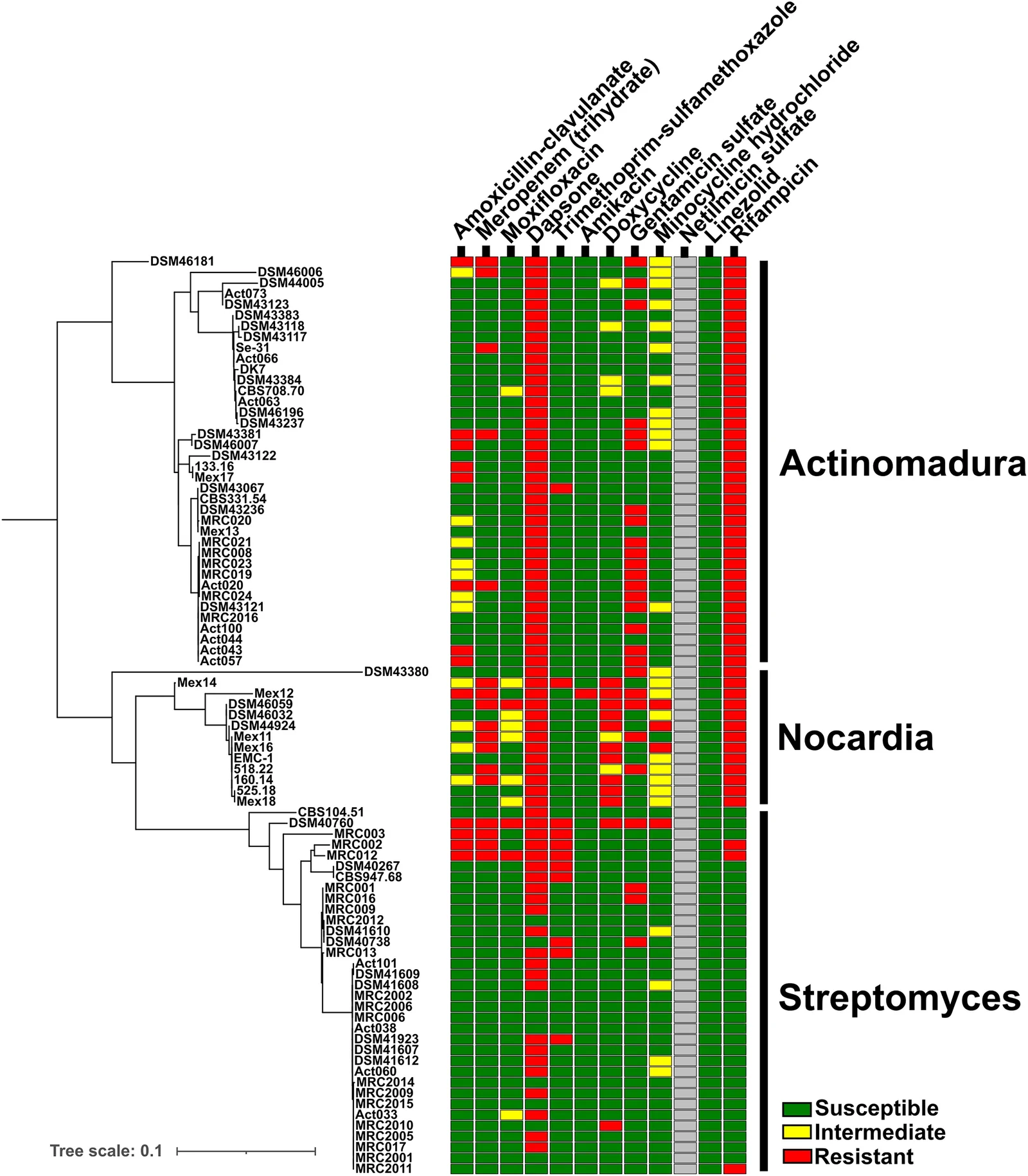
Antimicrobial susceptibility of *Actinomadura*, *Nocardia* and *Streptomyces* isolates against amoxicillin/clavulanate, meropenem, moxifloxacin, dapsone, trimethoprim/sulfamethoxazole, amikacin, doxycycline, gentamicin sulphate, minocycline, netilmicin, linezolid and rifampicin. Isolates with an MIC under the breakpoint were considered susceptible (green). Isolates with an MIC above the breakpoint were considered resistant (red). For some antibiotics also an intermediate category is indicated. Isolates with MICs in that range were considered intermediate (yellow). The phylogeny was constructed in iqtree2 from an alignment of 16S rRNA sequences.

In this study, the *in vitro* susceptibilities of 94 bacterial isolates associated with actinomycetoma were determined against 12 antibiotics. In Table 2, the MIC_50_ and range for each species and antibiotic are given. The individual MICs for each isolate can be found in Table S1. In Figure 1, it is indicated for each isolate whether they were susceptible (green), intermediate (yellow) or resistant (red) against the different antibiotics tested.

**Table 2.** MICs of the different antibiotics

		MIC_50_ (range), mg/L											
Organism	*n* isolates used	AMC	MEM	MXF	Dapsone	SXT	AMK	DOX	GEN	MIN	NET	LZD	RIF
*Actinocorallia weidayensis*	1	128/64	32	0.125	64	1/19	0.125	0.5	4	2	1	2	64
*Actinomadura cremea*	1	16/8	32	0.5	16	0.5/9.5	0.125	1	0.25	2	2	2	64
*Actinomadura geliboluensis*	2	32/16 (32/16–128/64)	4	0.5	64	0.5/9.5 (0.5/9.5–1/19)	0.125	1	8	2	2	2	64
*Actinomadura hallensis*	1	4/2	4	0.125	64	1/19	0.125	2	4	2	0.125	2	4
*Actinomadura latina*	1	1/0.5	0.25	0.125	64	2/38	0.125	1	1	1	0.125	2	64
*Actinomadura madurae*	20	4/2 (0.25/0.125–32/16)	4 (0.125–16)	0.125 (0.125–0.5)	64 (16–64)	0.5/9.5 (0.0625–16/304)	0.125	0.125 (0.125–0.5)	4 (0.25–8)	0.125 (0.125–2)	1 (0.125–1)	1 (0.25–2)	64 (8–64)
*Actinomadura meyerae*	2	64/32 (64/32)	4 (4–8)	0.125 (0.125–0.5)	64 (64)	1/19 (1/19)	0.125 (0.125)	0.125 (0.125)	0.25 (0.25)	0.125 (0.125–1)	0.125 (0.125)	2 (2)	64 (64)
*Actinomadura pelletieri*	12	1/0.5 (0.25/0.125–8/4)	4 (0.25–16)	0.125 (0.125–2)	64 (64)	0.0625/1.18 (0.0625/1.18–0.25/4.75)	0.125 (0.125)	1 (0.125–2)	0.25 (0.25–4)	1 (0.125–4)	0.125 (0.125–1)	1 (0.25–2)	64 (64)
*Actinomadura welshii*	2	0.25/0.125 (0.25/0.125–1/0.5)	4 (4)	0.125 (0.125)	32 (32)	0.25/4.75 (0.25/4.75)	0.125 (0.125)	0.5 (0.5–1)	1 (1–4)	0.5 (0.5–2)	1 (1–4)	1 (1–2)	8 (8–64)
*Nocardia asteroides*	1	64/32	2	2	32	0.5/9.5	0.25	2	1	4	1	2	2
*Nocardia brasiliensis*	13	8/4 (2/1–128/64)	16 (2–32)	2 (0.125–4)	64 (16–64)	0.5/9.5 (0.625/10.18–8/152)	1 (0.125–2)	8 (4–64)	1 (0.25–64)	4 (1–16)	1 (0.125–8)	4 (0.25–8)	64 (4–64)
*Nocardia farcinica*	1	16/8	32	2	64	8/152	1	16	1	4	2	8	64
*Nocardia mexicana*	1	64/32	32	1	8	1/19	64	16	64	4	16	4	64
*Nocardia vulneris*	2	4/2 (4/2)	8 (8–32)	2 (2–4)	64 (64)	0.25/4.75 (0.25/4.75–1/19)	1 (1–2)	8 (8)	0.25 (0.25–4)	4 (4–8)	2 (2–8)	1 (1–8)	16 (16–64)
*Streptomyces albidoflavus*	2	1/0.5 (1/0.5–4/2)	2 (2)	0.125 (0.125)	64 (64)	32/608 (32/608)	0.125 (0.125)	0.125 (0.125)	0.25 (0.25)	0.25 (0.25)	0.125 (0.125–0.25)	2 (2)	0.125 (0.125)
*Streptomyces albogriseolus*	1	128/64	64	1	64	8/152	0.125	1	0.25	0.125	0.125	4	64
*Streptomyces chrestomyceticus*	1	128/64	64	4	64	32/608	1	64	4	32	4	8	0.125
*Streptomyces griseus*	1	0.25/0.125	0.25	0.125	8	0.0625/1.18	0.125	0.125	0.25	1	0.125	1	0.125
*Streptomyces kordofanensis*	1	0.25/0.125	0.25	0.125	64	4/76	0.125	0.125	0.25	0.125	0.125	0.25	0.125
*Streptomyces radiopugnans*	1	32/16	16	0.5	32	16/304	0.125	1	0.25	0.125	1	4	0.125
*Streptomyces somaliensis*	7	1/0.5 (0.25/0.125–8/4)	0.25 (0.25–2)	0.125 (0.125)	8 (0.125–64)	2/38 (0.25/4.75–32/608)	0.125 (0.125)	0.5 (0.125–1)	1 (0.25–4)	0.25 (0.125–2)	2 (0.125–8)	2 (0.25–4)	0.125 (0.125–0.5)
*Streptomyces sudanensis*	19	2/1 (0.25/0.125–4/2)	1 (0.25–2)	0.125 (0.125)	8 (0.125–64)	0.5/9.5 (0.0625/1.18–4/76)	0.125 (0.125)	0.125 (0.125–8)	0.25 (0.25)	0.25 (0.125–2)	0.125 (0.125)	2 (1–4)	0.125 (0.125–4)
*Streptomyces werraensis*	1	128/64	64	4	64	16/304	0.125	1	0.25	0.125	0.125	4	64

AMC, amoxicillin/clavulanate; AMK, amikacin; DOX, doxycyline; GEN, gentamicin; LZD, linezolid; MEM, meropenem; MIN, minocycline; MXF, moxifloxacin; NET, netilmicin; RIF, rifampicin; SXT, trimethoprim/sulfamethoxazole.

As can be seen from Figure 1, the majority of the *Actinomadura*, *Nocardia* and *Streptomyces* species tested were susceptible to trimethoprim/sulfamethoxazole. Only 1 of 20 *A. madurae* isolates, 1 of 13 *N. brasiliensis*, 1 of 7 *S. somaliensis* and 1 of 19 *S. sudanensis* isolates were resistant to trimethoprim/sulfamethoxazole. However, *Nocardia farcinica* and seven from the remaining eight *Streptomyces* species, were resistant to trimethoprim/sulfamethoxazole. This indicated that 82 (87.2%) of the tested isolates were susceptible to trimethoprim/sulfamethoxazole. The actinomycetoma isolates were also highly susceptible to linezolid (100% susceptible) and amikacin (87.2% isolates susceptible).

Given that trimethoprim/sulfamethoxazole is one of the main treatments for actinomycetoma in the clinic, in combination with other antibiotics, we explored whether a common molecular basis for the observed resistance to this antibiotic could be identified. Previous reports have linked trimethoprim/sulfamethoxazole resistance in *N. farcinica* to the deletion of a specific region in the *folP2* gene.^26^ However, this region is intact in all three resistant isolates in which we identified the *folP2* gene. Furthermore, no other changes were identified that differentiated all resistant isolates from susceptible isolates in this region (Figure S1), including a previously reported alanine to valine substitution associated with resistance in *Nocardia.*^27^ Paralogue *folP* is the reported target of sulfamethoxazole, and the four trimethoprim/sulfamethoxazole-resistant *Streptomyces* isolates (*S. albidoflavus, S. albogriseoleus*, *S. radiopugnans* and *S. werraensis*) in which the gene was identified share several sequence variants that differ from susceptible isolates (Figure S2). However, none of these isolates are common actinomycetoma agents and are phylogenetically linked so it is difficult to draw strong conclusions on the relationship between these observed differences and trimethoprim/sulfamethoxazole resistance. The target of trimethoprim, *folA*, has very limited distribution amongst actinomycetoma agents in this study, and was identified only in *A. pelletieri*. All *A. pelletieri* isolates were susceptible to trimethoprim/sulfamethoxazole treatment. The *sul1* gene and the previously reported leucine to valine substitution in the *folC* gene were also not linked to trimethoprim/sulfamethoxazole resistance in our collection.^27,28^ The *sul1* gene was absent. All *Streptomyces* species, whether susceptible or resistant to trimethoprim/sulfamethoxazole, had valine at position 58 in the *folC* gene (relative to the *N. farcinica* reference sequence); also the two *Actinomadura* isolates with valine at position 58 in the *folC* gene were still susceptible to trimethoprim/sulfamethoxazole.

For the other antibiotics tested, species-specific differences in susceptibility patterns were noted. For amoxicillin/clavulanate, all *A. pelletieri*, *S. somaliensis* and *S. sudanensis* isolates were susceptible, with an MIC_50_ of 1/0.5, 0.25/0.125, and 2/1, respectively. However, both *Actinomadura meyerae* isolates, both *Actinomadura geliboluensis* isolates, 9 of 20 *A. madurae* isolates, 5 *N. brasiliensis* isolates, the *N. asteroides*, *N. farcinica*, *N. mexicana*, *S. albogriseolus*, *Streptomyces chrestomyceticus*, *S. werraensis* and *S. radiopugnans* isolates were either resistant or intermediate resistant (Table S1). The *Streptomyces* species generally had a single class A β-lactamase. *A. pelletieri* isolates also had one class A β-lactamase and one class D β-lactamase. Since the *Streptomyces* and *A. pelletieri* isolates were still susceptible, these β-lactamases seem not to be associated with resistance. The majority of *A. madurae* isolates, including the susceptible isolates, had two different class A β-lactamases, but none of these were specifically associated with resistant strains (Figure S3). For doxycycline, moxifloxacin and meropenem the *Streptomyces* and *Actinomadura* species were susceptible, while the percentages of resistance for *Nocardia* species were 30.7%, 21.2% and 38.3%, respectively. For minocycline some intermediate susceptibility was noted for *A. pelletieri*. For rifampicin, all *Actinomadura* and *Nocardia* species were resistant, while only 2 of 34 *Streptomyces* species were resistant. This difference in rifampicin resistance was quite striking and linked to differences in the rifampin resistance-determining region (RRDR) of *rpoB*^29^ between the susceptible *Streptomyces* species and the resistant *N. brasiliensis* and *Actinomadura* species (Data S1, Figure S4 and Figure S5).

## Discussion

The neglected subcutaneous tropical disease actinomycetoma can be caused by 40 different actinomycetes belonging to the genera *Actinomadura*, *Nocardia* and *Streptomyces.*^1^ Despite this large variation in causative agents, actinomycetoma cases are often treated with the same regimens. Here we demonstrate that there are substantial differences in susceptibility patterns between the most common causative agents of actinomycetoma, and that species identification and susceptibility testing should be performed before treatment is started.

As demonstrated, there was a large diversity in susceptibility patterns for the different actinomycetoma causative agents. The majority of the tested species were susceptible to trimethoprim/sulfamethoxazole, the cornerstone drug of the current actinomycetoma treatment regimens. Most *Nocardia* isolates (92%), *A. madurae* (95%), *A. pelletieri* (100%) and other *Actinomadura* species (100%) were susceptible to trimethoprim/sulfamethoxazole. This was similar to the 100% susceptibility reported by others for *N. brasiliensis.*^30,31^  *Streptomyces* species seemed to be less susceptible to trimethoprim/sulfamethoxazole, as only 85.7% of the *S. somaliensis* isolates, 95% of the *S. sudanensis* and 12.5% of the other *Streptomyces* species were susceptible to trimethoprim/sulfamethoxazole. Amikacin, the most commonly used drug in actinomycetoma combination treatment, showed activity against 98.9% of the causative agents studied here. The resistance to amikacin we noted for *N. mexicana* (MIC 64 mg/L), was in line with the resistance documented for three *N. mexicana* isolates by Rodríguez-Nava *et al*.^32^ Although not tested here, the combination trimethoprim/sulfamethoxazole/amikacin was found to be synergistic for other *Nocardia* species.^33^ Based on our results and the results published by others,^30,34–37^ the majority of actinomycetoma causative agents are susceptible to the standard trimethoprim/sulfamethoxazole/amikacin combination treatment used in Latin America.

In the Mycetoma Research Centre, trimethoprim/sulfamethoxazole is mostly combined with amoxicillin/clavulanate.^12^ For this antibiotic, species-specific differences in susceptibility were noted. For *S. somaliensis*, *S. sudanensis* and *A. pelletieri*, all tested isolates were susceptible to this drug. However, for *A. madurae* and *N. brasiliensis*, the heightened MIC_50_ and MIC_90_ values for amoxicillin/clavulanate indicated that resistance mechanisms may be emerging within these species. Most of the *A. madurae* isolates, including the susceptible isolates, encode two class A β-lactamases. As such, the patchy resistance of *A. madurae* to amoxicillin/clavulanate does not seem to correlate with the presence of a specific β-lactamase, but may be explained by mutations in either of the β-lactamase genes, or by alternative factors such as differences in drug efflux. Since isolates are still susceptible to trimethoprim/sulfamethoxazole it is not certain if this also will result in clinical resistance when combination treatment is used. Clinical evaluation studies are needed to determine this. Linezolid was recently introduced for the treatment of actinomycetoma. Interestingly, all actinomycetomas tested in this study showed 100% susceptibility to this antibiotic. For *A. madurae* and *N. brasiliensis*, this high susceptibility was also reported by others.^30,34^ Therefore, linezolid seems to be active against all actinomycetoma causative agents. This was also noted in the 16 patients treated with either linezolid alone or in combination with trimethoprim/sulfamethoxazole.^11,38^ Fourteen of 16 patients were cured within 1 to 6 months of treatment, one-third of the normal treatment time of 18 months often necessary in extensive actinomycetoma cases.^11,38^ The major drawback remains the cost of the drug, which might make it inaccessible for most actinomycetoma patients.^11^

For doxycycline, moxifloxacin, meropenem and rifampicin, species identification seems to be important before treatment is started. *A. madurae*, *A. pelletieri*, *S. somaliensis* and *S. sudanensis* showed high susceptibility rates to doxycycline, moxifloxacin and meropenem, but *N. brasiliensis* seemed to be less susceptible. Therefore, when actinomycetoma is caused by *N. brasiliensis,* other antibiotics might be more appropriate. Rifampicin could most likely only be used in the treatment of actinomycetoma caused by *S. somaliensis* and *S. sudanensis.* Only these two species seemed to be susceptible, while *A. madurae*, *A. pelletieri* and *N. brasiliensis* seemed to be intrinsically resistant. This resistance was most likely due to a combination of substitutions in the rifampicin-binding domain of RpoB and not the presence of a Rox gene in *Actinomadura* spp. and *N. brasiliensis* (Data S1). Despite this, once species identification has been performed, it might be cost-effective to switch *Streptomyces*-associated actinomycetoma patients to trimethoprim/sulfamethoxazole/rifampicin as it was 19 times more cost-effective than trimethoprim/sulfamethoxazole/amikacin^39^ and 63 times more cost-effective than using linezolid.^40^

In conclusion, our findings highlight a variation in susceptibility across the species studied. This species variability underscores that MIC testing and/or species-level identification is essential for effective therapy. While these *in vitro* results provide a necessary foundation, they are limited by the absence of host–pathogen and drug dynamics. Consequently, the next step is to correlate these MIC values with clinical outcomes to determine if this laboratory-observed resistance translates into treatment failure in practice.

## Supplementary Material

dkag325_Supplementary_Data

## Supplementary data

Figures S1–S5, Tables S1 and S2, and Data S1 are available as Supplementary data at *JAC* Online.
