# Supplementary material for: Potential clinical implications due to essential differences in antimicrobial susceptibility profiles of actinomycetoma causative agents of the Actinomadura, Nocardia and Streptomyces genera towards commonly used anti-actinomycetoma drugs
Source: J Antimicrob Chemother. 2026 Sep 25;81(10):dkag325. doi: 10.1093/jac/dkag325 (PMC13613230; doi:10.1093/jac/dkag325)
Supplement: Supplementary file 1 [file dkag325_supplementary_data.zip › Supplementary data.docx]

**Supplementary data**

**Content**

[Supplementary Figure 1: Conservation of the region of folP2 associated with trimethoprim-sulfamethoxazole resistance	1](#_Toc239681559)

[Supplementary Figure 2: Regions of folP with conserved differences between trimethoprim-sulfamethoxazole resistant and susceptible Streptomyces isolates within our dataset.	2](#_Toc239681560)

[Supplementary Figure 3: A phylogeny of all beta-lactamase genes identified by amrFinderPlus in the genomes of actinomycetoma agents	3](#_Toc239681561)

[Supplementary data 1: differences in the rifampin resistance-determining region of *rpo*B associated with rifampicin resistance in *Actinomadura*	4](#_Toc239681562)

[Supplementary Figure 4: Molecular basis of rifampicin resistance in *Actinomadura* species and *Nocardia brasiliensis.*	5](#_Toc239681563)

[Supplementary Figure 5: Rox alone is not predictive of Rifampicin resistance	6](#_Toc239681564)

# **Supplementary Figure 1: Conservation of the region of folP2 associated with trimethoprim-sulfamethoxazole resistance**

**Supplementary Figure 1:** Conservation of the region of folP2 associated with trimethoprim-sulfamethoxazole resistance in both susceptible and resistant strains collected in our dataset. The x-axis indicates the amino acid position of the homologous sequence in *N. farcinica*. This region was previously found to be deleted in trimethoprim-susceptible and resistant isolates in our dataset.

# **Supplementary Figure 2: Regions of folP with conserved differences between trimethoprim-sulfamethoxazole resistant and susceptible Streptomyces isolates within our dataset.**

**Supplementary Figure 2:** Regions of folP with conserved differences between trimethoprim-sulfamethoxazole resistant and susceptible *Streptomyces* isolates within our dataset. The x-axis indicates the amino acid position of the homologous sequence in *N. farcinica*.

# **Supplementary Figure 3: A phylogeny of all beta-lactamase genes identified by amrFinderPlus in the genomes of actinomycetoma agents**

**Supplementary Figure 3**: A phylogeny of all beta-lactamase genes identified by amrFinderPlus in the genomes of actinomycetoma agents, inferred using iqtree2 and visualised in iToL. Amoxicillin-clavulanic acid susceptible, intermediate, and resistant strains are labelled in green, yellow and red respectively. Red dots on branches indicate ultrafast boostrap support >95%. Two reference class A beta-lactamases from *Nocardia brasiliensis* and *Nocardia asteroides* are indicated in black

# **Supplementary data 1: differences in the rifampin resistance-determining region of *rpo*B associated with rifampicin resistance in *Actinomadura***

The clear difference in rifampicin resistance was quite striking and therefore we investigated the genomes of isolates for a potential explanation. For *Nocardia* species several routes to rifampicin resistance have been described: either point mutations in the rifampicin binding domains of RNA polymerase β subunit (*rpoB*), the presence of a second RpoB gene (*rpoB2*)^38^ or the presence of the *rox* gene encoding for a rifamipicin monooxygenase converting rifampicin into the non-active 2’-N-hydroxy-4-oxo-rifmapicin.^39^ As can be seen in **Supplementary Figure 4A,** there are some clear differences in the Rifampin Resistance-Determining Region (RRDR) of *rpoB*^40^ between the susceptible *Streptomyces* species and the resistant *Nocardia brasiliensis* and *Actinomadura* species. From these the amino-acid substitutions at position 518 (*Streptomyces* spp have Asparagine (N), *Actinomadura* spp have threonine (T) or serine (S) and *N. brasiliensis* has aspartic acid (D)) and position 522 (*Streptomyces* spp have S, *Actinomadura* spp have alanine (A) or D and *N. brasiliensis* has A or S) were most likely associated with the identified resistance (**Supplementary Figure 4B).***^38^* A potential duplication of the RpoB gene was found in the common ancestor of *A. madurae* isolates, but not *Actinomadura* as a whole (**Supplementary Figure 4C**). A gene with homology to the *Rox* gene was found in all three genera, and its presence or absence did not seem to correlate with rifampicin resistance (**Supplementary Figure 5)***.* Thus, the observed resistance was most likely due to a combination of substitutions in the rifampicin binding domain of RpoB and not the presence of a Rox gene in *Actinomadura* spp and *N. brasiliensis*. ^38, 39^

# **Supplementary Figure 4: Molecular basis of rifampicin resistance in *Actinomadura* species and *Nocardia brasiliensis.***

**Supplementary Figure 4:** Molecular basis of rifampicin resistance in *Actinomadura* species and *Nocardia brasiliensis.* **A**. Protein sequence of the RRDR of the RpoB gene with differences in amino acids highlighted. Amino acid positions reported are relative to *E. coli* RpoB reference sequence (UniProt: P0A8V2). **B.** AlphaFold structure of the *Streptomyces* RpoB protein (yellow) aligned against an existing crystal structure of the *E. coli* RNA polymerase RpoB S531L complex (cyan) with rifampin (PDB: 5UAL), highlighting the residues where we see differences between *Streptomyces* and *Actinomadura* and *Nocardia* in red indicating that these residues are associated with rifampicin binding. **C.** A phylogeny of homologs of the RpoB gene within *Actinomadura* and *Streptomyces* from susceptible isolates (green) and resistant isolates (red).

# **Supplementary Figure 5: Rox alone is not predictive of Rifampicin resistance**

**Supplementary Figure 5**: **Rox alone is not predictive of Rifampicin resistance.** A phylogeny of ROX related genes in Actinomadura and Streptomyces isolates. Red dots on branches indicate >95% bootstrap support. Both Rifampacin resistant (red) and susceptible (green) isolates encode a variety of genes closely related to characterised ROX from *Nocardia farcinica* and *Streptomyces venezuelae* (black).
